# Digital Planning to Enhance Diagnosis and Precision in Correcting Excessive Gingival Display in the Presence of Asymmetrical Maxillary Position: A Case Report

**DOI:** 10.1055/s-0044-1785535

**Published:** 2024-05-14

**Authors:** Isabella Neme Ribeiro Reis, Gabriel Bittencourt Damin, Camilla Rodrigues Pereira, Matheus de Alencar Ichigi, Luiza Orsi Caminha Sant'Anna, Rubens Spin-Neto, Giuseppe Alexandre Romito

**Affiliations:** 1 Division of Periodontics, Department of Stomatology, School of Dentistry, University of São Paulo, São Paulo, Brazil; 2 Department of Dentistry and Oral Health, Section for Oral Radiology, School of Dentistry, Aarhus University, Aarhus, Denmark

**Keywords:** gummy smile, excessive gingival display, aesthetics, digital planning, digital smile design

## Abstract

This case report addresses the treatment of excessive gingival display (EGD) in the context of maxillary asymmetry, zenith irregularities, and occlusal plane inclination. Digital planning was pivotal in formulating a precise treatment strategy by incorporating facial photographs, digital models, and cone-beam computed tomography data. Parameters, including occlusal plane inclination, teeth position, and lip-to-gingival margin relationships, were considered to ensure treatment alignment with the patient's facial characteristics. Notably, during the planning phase, it was evident that the conventional approach using the cementoenamel junction as the apical limit for incisions would result in asymmetry. Consequently, the gingival margin position was defined in accordance with facial and lip features. The chosen treatment, flapless crown lengthening, was tailored to the patient's thin phenotype and guided by measurements derived from digital planning. Postsurgery, the patient experienced a swift and painless recovery. A harmonious smile with a stable gingival margin position was achieved at the 1-year follow-up, seamlessly complementing the patient's facial attributes. This case underscores the importance of personalized EGD treatment and the value of digital planning in enhancing diagnostic accuracy and precise treatment planning, ultimately facilitating optimal treatment strategies.

## Introduction

The relationship between teeth and the surrounding gingival tissue significantly impacts the aesthetics of a smile. Society has set specific standards for what constitutes an attractive or unattractive smile, focusing on factors like the symmetry of teeth, their positioning, and the amount of gingival tissue visible when one smiles. 1 2 3 This condition, often called excessive gingival display (EGD) or a “gummy smile,” is a common issue in dental aesthetics. Specifically, it is characterized by more than 3 mm of gingiva exposure during smiling. 4

EGD can have a detrimental impact on a patient's self-perception, self-esteem, social interactions, and professional relationships. A study by Malkinson et al found that the degree of gum exposure was inversely correlated with perceptions of friendliness, trustworthiness, intelligence, and self-confidence. 3 Additionally, a study among a young population revealed that EGD negatively affected individuals' quality of life. On the other hand, corrective procedures were shown to significantly enhance patients' self-esteem and confidence when smiling and engaging in social interactions. 2

In most cases, a surgical approach is indicated for treating EGD, with the procedure's invasiveness varying based on the underlying causes and characteristics of the case. 5 6 One common cause of EGD is altered passive eruption (APE). APE refers to the failure of the gingival margin to reach and stabilize at the cementoenamel junction (CEJ) during dental eruption, resulting in the concealed cervical portion of the anatomical crown. 7 8 The specific type of APE determines the appropriate surgical technique to be employed, 5 6 7 and an accurate diagnosis relies on a comprehensive clinical examination, which considers the relationship and position of the CEJ, mucogingival junction, and alveolar bone crest. 5 6 7 Diagnosing the condition can be challenging when other factors, such as facial and maxillary asymmetries and asymmetric zeniths, coexist with EGD in cases of a gummy smile. A comprehensive diagnosis should include an analysis of the face, lips, teeth, and gingival tissue. 9 10

Digital planning has emerged as a highly effective approach to address challenges related to diagnosis and enhance predictability and safety in esthetic procedures. 11 12 By utilizing this method, clinicians can generate three-dimensional (3D) models of a patient's teeth and gingival tissues, which can then be superimposed with a cone-beam computed tomography (CBCT) scan and a facial photo or scan. 11 This comprehensive evaluation enables the simultaneous assessment of teeth, gingival tissues, bone levels and architecture, as well as their relationship with the lips and face. Consequently, a more precise evaluation of the relevant anatomical structures can be achieved, ensuring a harmonious outcome that aligns with the patient's facial features and skeletal aspects. 9

This case report showcases digital planning to devise a comprehensive treatment strategy for EGD caused by APE, accompanied by asymmetric maxilla, gingival zeniths, and an inclined occlusal plane. By superimposing the patient's facial photograph, digital models, and CBCT file, a precise surgical plan was developed to achieve an aesthetically balanced smile in harmony with the patient's facial characteristics.

## Case Report

### Diagnosis and Planning

A 21-year-old female patient presented to the School of Dentistry at the University of São Paulo with a chief complaint of EGD when smiling and asymmetric gingival position. The patient had completed orthodontic treatment, and her teeth alignment and position were adequate and met her expectations. Periodontal assessment revealed a healthy periodontal condition and thin gingival phenotype.

**Fig. 1:**
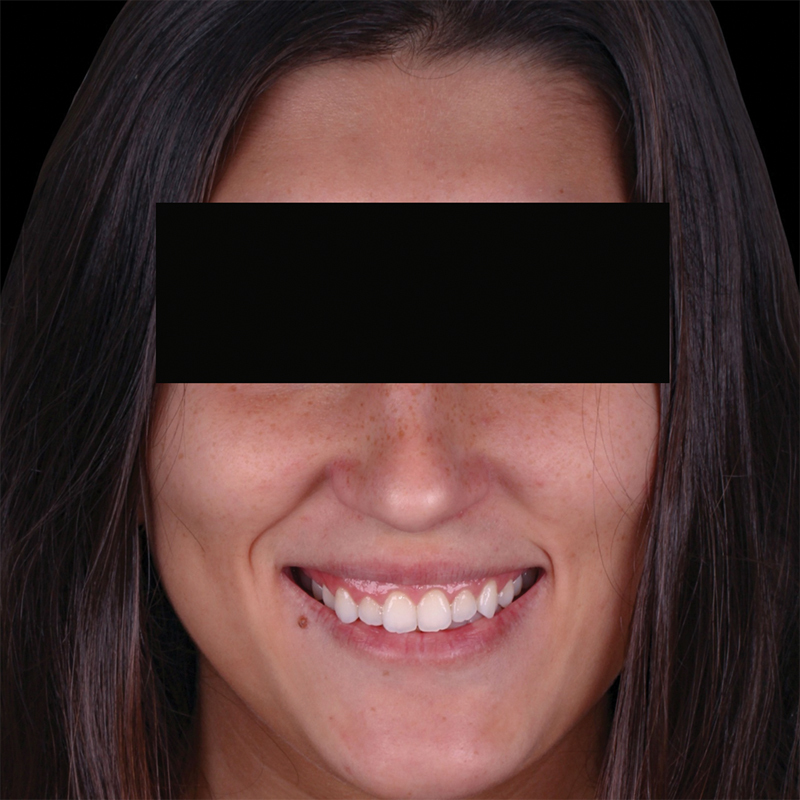
Extraoral photograph illustrating excessive gingival display.

**Fig. 2:**
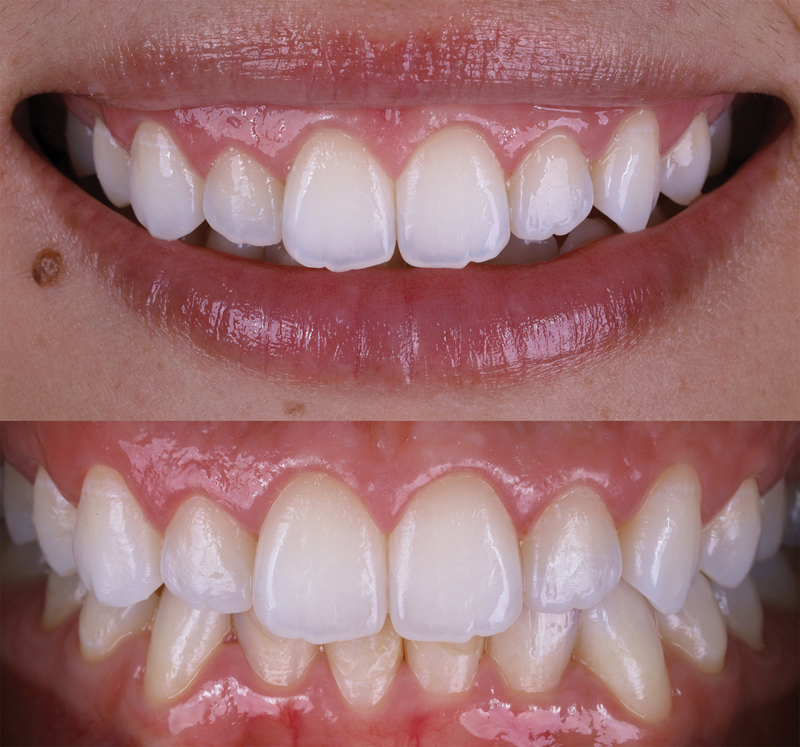
Initial intraoral photograph depicting excessive gingival display.

The clinical examination confirmed the presence of EGD attributed to APE. The mucogingival junction was located apical to the CEJ, and the bone crest was located at the CEJ level. This condition is characterized as APE type 1 subgroup B ( Figs. 1 and 2 ). 7 8

Intraoral and extraoral photographs were taken, and both dental arches were scanned using an intraoral scanner (Trios 4, 3Shape, Denmark) to generate 3D images in standard tessellation language (STL) format. Furthermore, CBCT was performed with lip retractors (Maquira, Paraná, Brazil) to assess the dimensions of the gingival tissue concerning the underlying bone structure. These files were then imported into a CAD software (Exocad DentalCAD 3.0, Exocad Gmbh) to facilitate superimposition of the facial photos, digital models, and CBCT files for virtual smile planning.

The superimposition of the facial photo with the digital files in DICOM and STL formats allowed clear visualization of dental and osseous structures, as well as accurate positioning of soft tissues and their relationship with the lips and face. 12 The CBCT file offered information on gingival thickness and the relationship between the gingival margin and the osseous crest in the case. 13

**Fig. 3:**
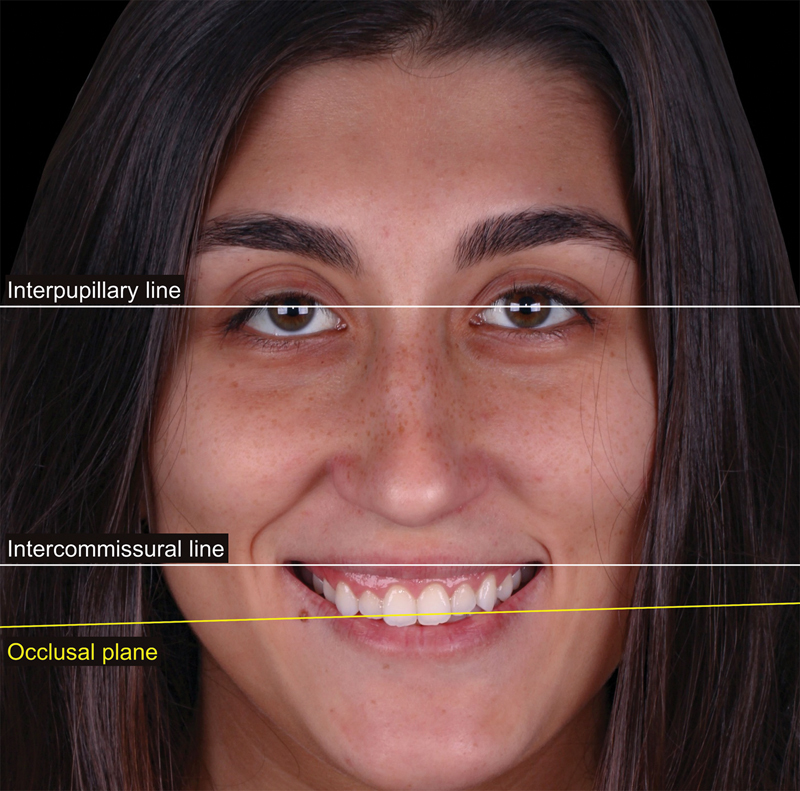
Extraoral photograph highlighting the inclination of the occlusal plane (maxillary asymmetry) and variations in gingival exposure between the right and left sides.

The virtual smile planning considered individualized dimension parameters such as smile height and curvature. Horizontal reference lines were established based on the interpupillary and intercommissural lines. 13 14 The comprehensive digital analysis revealed an asymmetric maxillary position and tilted occlusal plane, characterized by greater exposure of the maxilla on the right side ( Fig. 3 ).

**Fig. 4:**
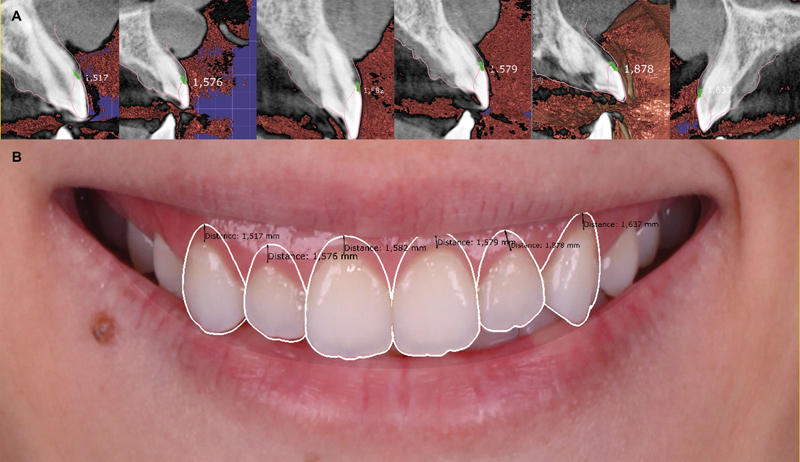
Measurements from the gingival margin to cementoenamel junction for each tooth ( **A** ). Transposition of these measurements onto the initial smile photograph, anticipating the expected results by following this reference ( **B** ).

**Fig. 5:**
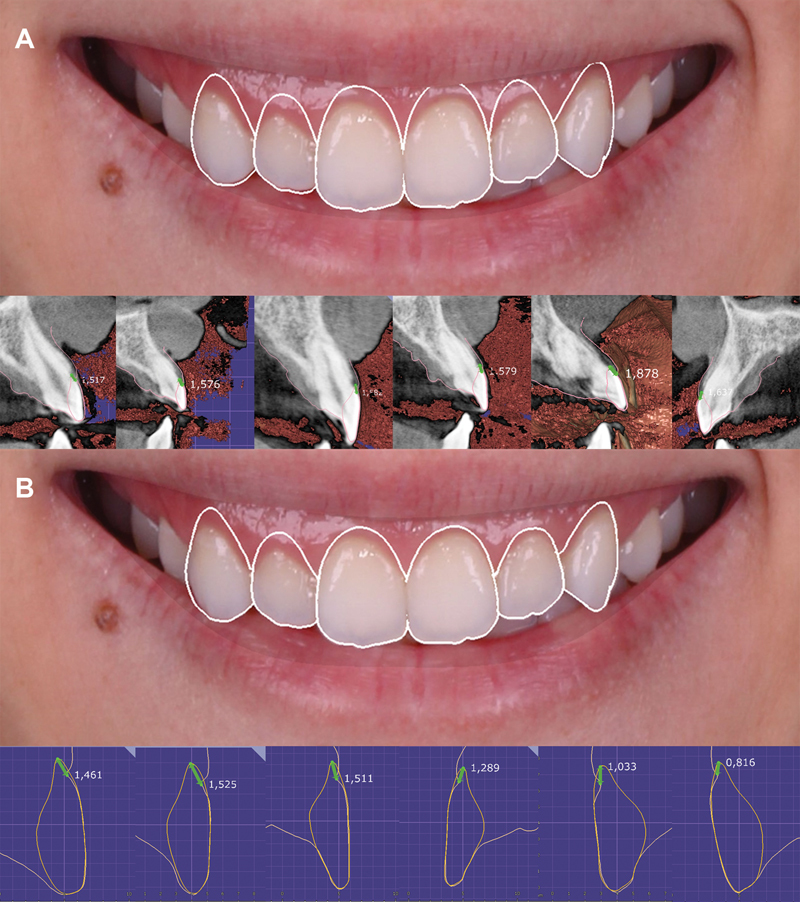
Treatment planning based on the cementoenamel junction as the reference for incisions ( **A** ). Treatment planning adjusted to better align with facial and lip features ( **B** ).

**Fig. 6:**
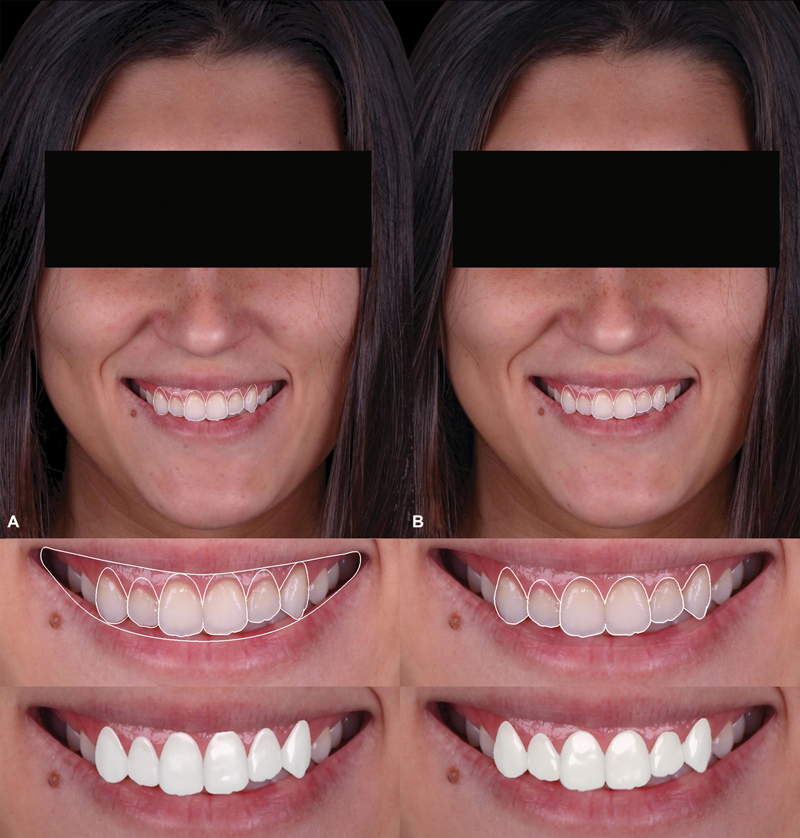
Representation of the differences between approaches using the cementoenamel junction as the apical limit ( **A** ) and those considering facial and lip features ( **B** ).

Subsequently, a dentogingival analysis was performed to evaluate the distance between the upper lip and the CEJ, specifically focusing on gingival exposure. This analysis included drawing a line along the inner part of the lips and analyzing the distance between the line of the superior lip and the CEJ of each tooth (#13, #12, #11, #21, #22, and #23) ( Fig. 4A ). The measurements indicated that a greater amount of gingival exposure would occur on the right side when considering the CEJ as the reference for the postsurgical gingival margin. Consequently, it was determined that using the CEJ as the apical limit for incisions would not be suitable ( Figs. 4B and 5A ). Instead, the planning for future gingival margins was meticulously adjusted to ensure that the margins of teeth #21, #22, and #23 would align with those of #11, #12, and #13 ( Fig. 5B ). As a result, the future gingival margin would be positioned in a more coronal orientation in relation to the CEJ ( Fig. 5B ). This strategic alignment aimed to achieve harmony with the lip and facial features, ensuring consistent distances between the gingival margin and the lip line across homologous teeth ( Fig. 6 ). Moreover, when assessing the position of the postsurgical gingival margin in relation to the bone crest, a precise determination of the necessary osteotomy was possible. This consideration influenced the choice of a flapless surgical technique, as for the specific case, only a minimal reduction in bone height and no reduction in bone width were required.

To ensure accurate translation of the measurements obtained in the software to the patient's gingival tissue, a calibration process was undertaken using the real length of the teeth crowns. This calibration enabled precise transfer of the measurements from the software to the patient's gingival tissue, facilitating meticulous planning and precise execution of the incisions.

### Surgical Procedure

The chosen surgical technique for this case was flapless crown lengthening, which does not involve the elevation of a mucoperiosteal flap. This decision was based on the patient's thin phenotype and no need for bone width reduction, as observed through clinical examination and CBCT evaluation.

The procedure began by administering anesthesia with 4% Articaine 1:100,000 (DFL, Brazil) to the treatment area. Incisions were meticulously executed according to the measurements obtained from the digital planning. These incisions were carefully marked and made with an internal bevel, utilizing a number 15C scalpel blade (Swann-Morton, England).

**Fig. 7:**
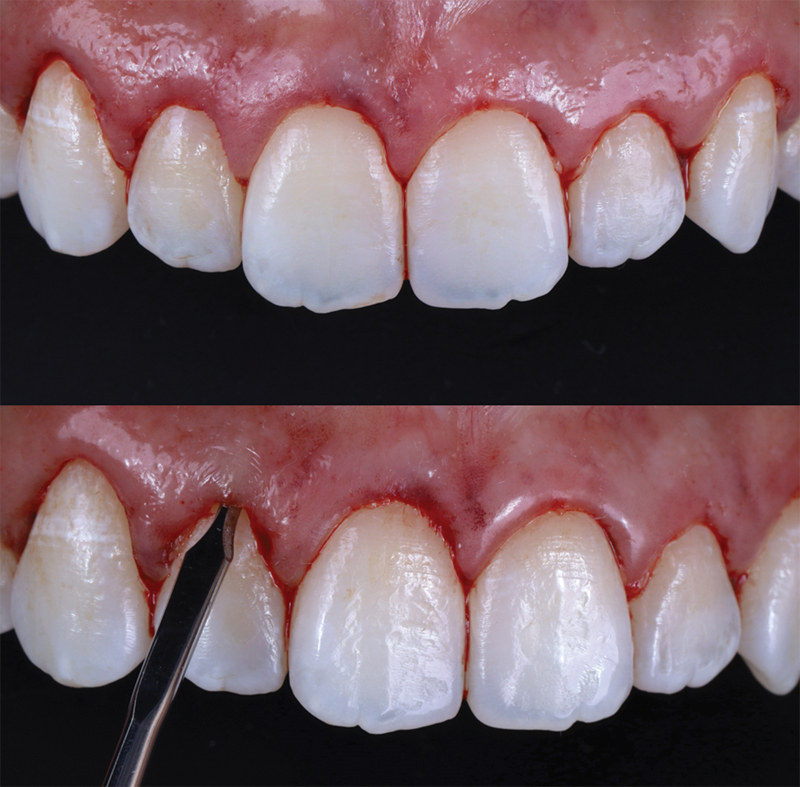
Gingivectomy and flapless osteotomy procedures.

After the incisions, a gentle tissue detachment process was conducted using a Molt tissue elevator (Hu-Friedy, Germany), with the utmost care taken to preserve the integrity of the papillae. To reestablish the desired supracrestal tissue attachment space concerning the postsurgical gingival margin, an osteotomy was performed using a Micro Ochsenbein Chisel (Quinelato, Brazil) ( Fig. 7A ). Since this technique is minimally invasive and does not require a mucoperiosteal flap, sutures were not necessary.

Postoperatively, the patient received comprehensive instructions, which included the use of a 0.12% chlorhexidine digluconate mouthwash (Periogard, Colgate, Brazil) for 7 days and analgesics containing 500mg of dipyrone for 3 days to manage any discomfort. The immediate postoperative assessment revealed harmonious gingival margin positions ( Fig. 7B ).

During the postoperative period, the patient exhibited excellent healing of the gingival tissue, with no adverse effects. Furthermore, the patient reported a swift and painless recovery.

**Fig. 8:**
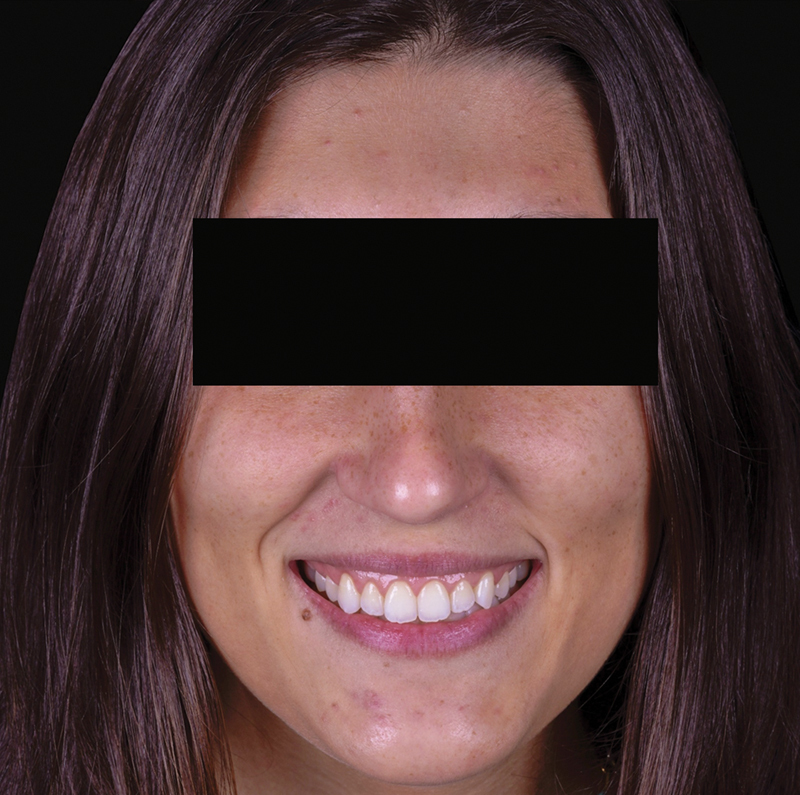
Extraoral photograph showing the postoperative healing process 1 year after the procedure.

**Fig. 9:**
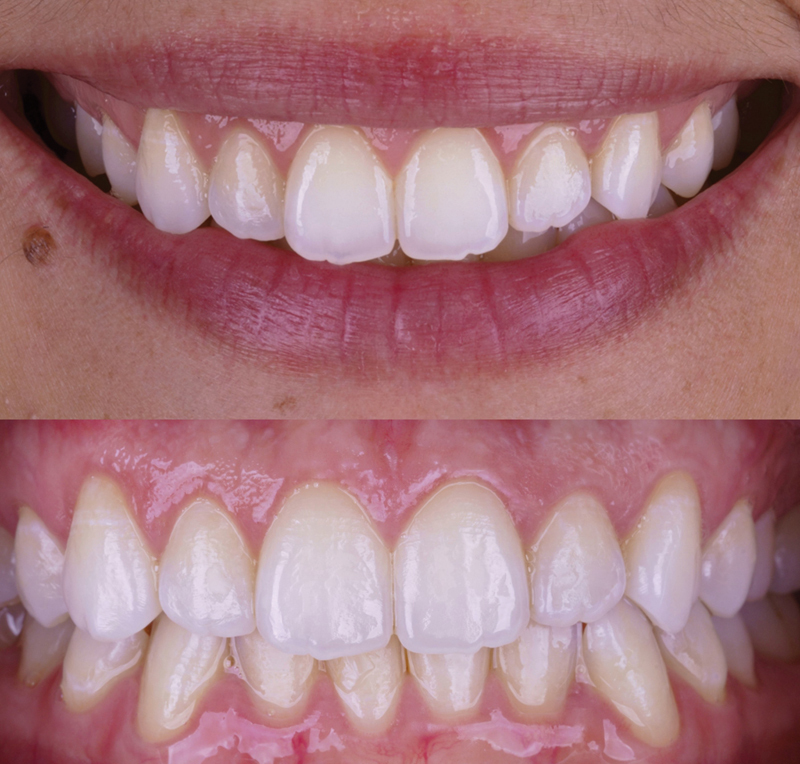
Intraoral photograph taken during the 1-year follow-up examination.

**Fig. 10:**
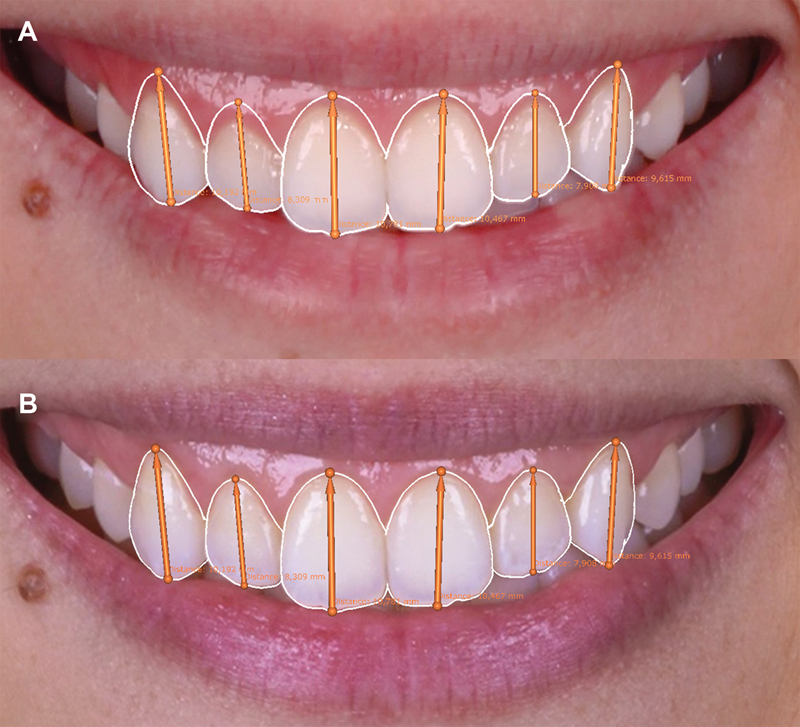
Correspondence between digital planning ( **A** ) and the postsurgery outcome 1 year later ( **B** ).

At the 1-year follow-up appointment, a harmonious smile was observed, along with a stable position of the gingival margin ( Figs. 8 and 9 ). This result closely aligned with the parameters established in the digital planning phase ( Fig. 10 ). The professionals involved and the patient were satisfied with the esthetic and functional outcomes they achieved.

## Discussion

This case report underscores the importance of tailoring the treatment of EGD to each patient's unique features and highlights the advantages of employing comprehensive digital planning. The digital planning revealed critical aspects of the patient's facial characteristics, occlusal plane inclination, teeth, and their respective gingival margin positions and underlying bone. 15 16 This information facilitated the development of a personalized treatment plan to provide the patient with the best possible outcome while considering their facial features, resulting in a highly satisfactory result. 15 16

Key facial elements directly associated with smile esthetics treatment include evaluating the facial thirds and aligning the bipupillary line with the line passing through the commissures and the occlusal plane. 9 14 17 Apart from facial elements, other important factors to consider during the planning process include the length and curvature of the lips, upper lip contraction, symmetry of the mouth corner, gingival contour, and zenith position. 14 18 These elements collectively contribute to a comprehensive analysis and planning for achieving optimal results.

Digital planning played a pivotal role in evaluating the relationship between the CEJ and the gingival margin and its impact on the final esthetic outcome. The analysis revealed that relying on the CEJ as a reference for the incisions would result in asymmetrical positioning of the gingival zeniths concerning the upper lip and face. Therefore, the decision was made to deviate from using the CEJ as a reference and instead consider the distance between the gingival margin and the lips in a way that ensured harmonious alignment between the right and left sides. This approach resulted in an aesthetically pleasing outcome while also ensuring the precise removal of gingival and bone tissue.

Gateño et al 19 conducted a study on aesthetic skeletal variations in the population using mirroring and superimposing techniques at specific facial landmarks in the context of maxillary and occlusal plane asymmetry. The authors found that skeletal asymmetry can arise from various factors, including congenital conditions like hemifacial microsomia, environmental triggers such as trauma, infection, or tumors, and functional influences like habits or occlusal interference. These asymmetries can exhibit distinct patterns influenced by individual characteristics, onset timing, muscular compensatory mechanisms, and facial musculoskeletal development. However, classifying the asymmetry remains challenging due to the complex interplay of these variables. 20 In the presented case, the digital planning revealed a noticeable maxillary asymmetry, leading to a modification in the treatment planning. 19

Digital planning proved invaluable for achieving more accurate diagnoses, offering multiple advantages, including precise treatment planning, improved communication, visualization of the final outcome, reduced error risks, and time-saving benefits. 21 22 23 This approach allows for comprehensive facial analysis, taking into account clinical factors that may be overlooked during a conventional visual examination. 21 22 23 In the case presented, digital planning played a critical role in determining the optimal position for the postsurgical gingival margin. It also provided the necessary measurements to address anatomical concerns while ensuring optimal supracrestal insertion and achieving the desired aesthetic results. 12 17 23

This clinical case report aligns with previous studies that emphasize the advantages of employing digital planning in EGD treatment to enhance aesthetics. 9 14 21 22 Particularly, the superimposition of the facial photograph with STL and CBCT files allowed an expended view of the case, to comprehensively assess all aspects of the patient's face. The integration of digital planning broadens the diagnostic perspective, allowing for the formulation of optimal treatment plans while benefiting from the aforementioned advantages.

## Conclusion

Digital planning played a pivotal role in achieving precise diagnosis and treatment for EGD in a case characterized by APE with coexisting factors, such as asymmetric maxillary position. Through the integration of intraoral digital models, face photos, and CBCT scan, a comprehensive assessment encompassed teeth, gingival tissues, bone structure, and their interaction with lips and facial features. The traditional approach, which relies on the CEJ as the apical limit for incisions, risked causing asymmetry and aesthetic disharmony in this specific case. Digital planning facilitated the development of an individualized treatment strategy that considered the postsurgical gingival margin's alignment with the patient's lips and facial features. This approach proved indispensable for achieving more accurate diagnoses in cases complicated by asymmetry, ultimately optimizing both functional and aesthetic outcomes in EGD treatment.
